# Positron emission tomography in cardiac amyloidosis: current evidence and future directions

**DOI:** 10.1007/s10741-025-10493-3

**Published:** 2025-02-10

**Authors:** Alberto Aimo, Yu Fu Ferrari Chen, Vincenzo Castiglione, Claudio Passino, Dario Genovesi, Assuero Giorgetti, Michele Emdin, Giuseppe Vergaro

**Affiliations:** 1 Health Sciences Interdisciplinary Center, Scuola Superiore Sant’Anna, Via G. Moruzzi 1, 56124 Pisa, Italy; 2 Fondazione Toscana Gabriele Monasterio, Via G. Moruzzi 1, 56124 Pisa, Italy

**Keywords:** Positron emission tomography, PET, Cardiac amyloidosis, Amyloid tracers, Nuclear medicine, Diagnosis, Prognosis, Multimodal imaging

## Abstract

The increasing recognition of cardiac amyloidosis (CA) as a cause of heart failure, coupled with advancements in therapeutic options, has underscored the need for early detection. Positron emission tomography (PET) imaging emerged as a promising non-invasive tool for diagnosing and managing CA. This review provides a comprehensive analysis of current PET imaging techniques, focusing on radiotracers, including [^11^C]Pittsburgh Compound B, [^18^F]Flutemetamol, [^18^F]Florbetapir, [^18^F]Florbetaben, [^18^F]-sodium fluoride, and [^124^I]Evuzamitide. PET imaging’s ability to differentiating CA subtypes and quantify amyloid burden contributes defining prognosis and aids in monitoring treatment response. However, standardizing imaging protocols and establishing definitive diagnostic thresholds remain challenging. As PET imaging continues to evolve, it promises to improve patient outcomes by facilitating earlier diagnosis, more accurate subtype differentiation, and better treatment monitoring in CA.

## Introduction

Amyloidosis is an infiltrative disorder resulting from the extracellular accumulation of aggregates formed by misfolded proteins leading to organ damage [1]. This condition manifests in various forms based on the amyloidogenic protein involved. Cardiac amyloidosis (CA) usually arises from tissue accumulation of transthyretin (either wild-type—ATTRwt—or mutated/variant—ATTRv) or immunoglobulin light-chains (AL). In much rarer cases, the heart may be involved in the context of reactive (AA) or leucocyte chemotactic factor 2 (ALECT2) amyloidosis, predominantly affecting the liver and kidney, not subjected to PET imaging studies to date. An early etiological diagnosis can lead to the initiation of disease-modifying treatments, potentially improving patient outcomes [1].

Nuclear medicine has always been crucial to diagnose CA. Initially, radioisotope ventriculography identified morphological abnormalities in CA, then specific tracers revealed abnormalities in myocardial perfusion [2], metabolism [3], and innervation [4, 5] associated with amyloid accumulation. Yet, the most significant contribution of nuclear medicine in CA is the use of bone tracers labeled with metastable technetium 99, such as [^99m^Tc]Tc-pyrophosphate ([^99m^Tc]Tc-PYP), [^99m^Tc]Tc-3,3-diphosphono-1,2-propanodicarboxylic acid ([^99m^Tc]Tc-DPD), and [^99m^Tc]Tc-hydroxymethylene diphosphonate ([^99m^Tc]Tc-HMDP). These radiotracers exhibit high affinity for ATTR deposits, except for a few *TTR* variants [6–8]. Cardiac uptake can be semi-quantitatively evaluated using the Perugini scale [8]. In the absence of a monoclonal protein, a cardiac uptake at least as intense as the bone (corresponding to Perugini grades 2 or 3, respectively) allows diagnosing ATTR-CA with no need for histological confirmation. When a monoclonal protein is present, tissue biopsy is required for a definite diagnosis of CA [9, 10].

Positron emission tomography (PET) is a nuclear imaging technique employing tracers attached to radionuclides undergoing positron emission decay. The interaction between a positron and an electron results in the annihilation of both particles, producing two photons traveling in opposite directions, detectable by specialized scanners. PET imaging has found numerous clinical applications due to the variety of tracers available for specific metabolic processes. Furthermore, hybrid PET/computed tomography (CT) imaging has enabled accurate anatomical localization of areas with radiotracer uptake [11]. The development of PET tracers for detecting β-amyloid deposits in Alzheimer’s disease (AD) and their application to nuclear cardiology have opened new perspectives for the non-invasive diagnosis of CA [11, 12]. To date, given the potential function of quantification amyloid burden, the emerging prognostic role of PET should encourage researchers to fill the lack of evidence about follow-up and response to therapies.

For this review, pertinent studies were searched in PubMed/Medline (updated December 2024) using the following terms: *positron emission tomography*, *PET tracers*, *cardiac amyloidosis*,* [*^*11*^*C]Pittsburgh Compound B*,* [*^*18*^*F]Flutemetamol*,* [*^*18*^*F]Florbetapir*,* [*^*18*^*F]Florbetaben*,* [*^*18*^*F]-sodium fluoride*, and* [*^*124*^*I]Evuzamitide*. Given the design of this work as a narrative review, no formal criteria for study selection or appraisal were enforced.

## PET tracers

### ^11^C-Pittsburgh compound B

#### Chemical structure, half-life, and FDA approval

**Fig. 1:**
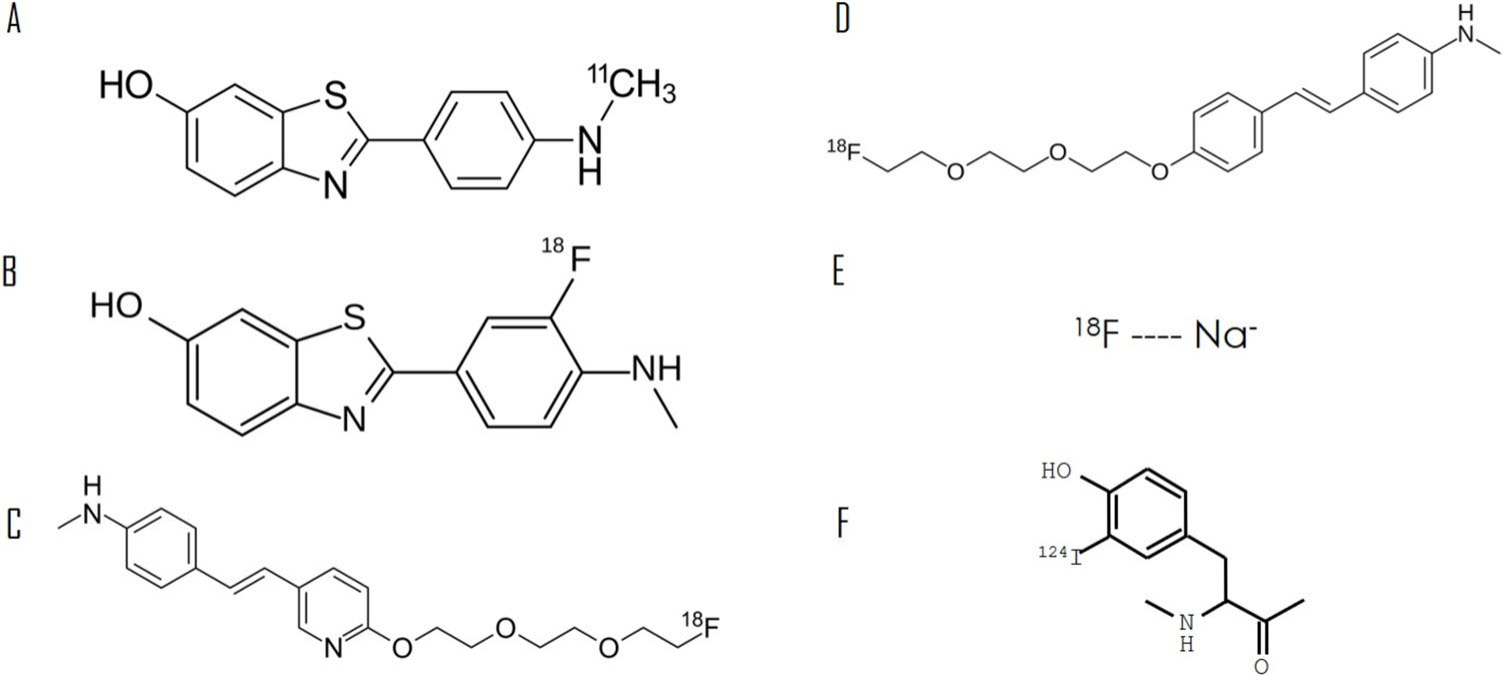
Molecular structures of various amyloid radiotracers. **A** [^11^C]Pittsburgh compound B by Ed (Edgar181)—own work, public domain, https://commons.wikimedia.org/w/index.php?curid=65798106. **B** [^18^F]Flutemetamol by Anypodetos—own work, CC0, https://commons.wikimedia.org/w/index.php?curid=35531313. **C** [^18^F]Florbetapir by Ed (Edgar181)—own work, public domain, https://commons.wikimedia.org/w/index.php?curid=21718327. **D** [^18^F]Florbetaben by Ed (Edgar181)—own work, public domain, https://commons.wikimedia.org/w/index.php?curid=31776306. **E** ^18^F-sodium fluoride. **F** [^124^I]Evuzamitide: the alpha helix with charged lysine side chains

[^11^C]PiB is a radioactive analog of thioflavin T (Fig. 1). Given the short half-life of 20.4 min, the primary limitation of [^11^C]PiB PET is the requirement for an on-site cyclotron to produce the tracer [13]. This tracer has not been approved by the FDA.

### Diagnosis

It enables detection and quantification of β-amyloid deposits in AD [14, 15]. A pioneering study in 2013 demonstrated that [^11^C]PiB imaging could also detect CA. The authors observed myocardial [^11^C]PiB uptake in 10 patients with either AL- or ATTR-CA, with no uptake in 5 healthy controls [16]. Subsequent case–control studies have confirmed the specificity of [^11^C]PiB uptake in CA. For instance, a study involving 22 patients with monoclonal gammopathy showed [^11^C]PiB uptake in 13 out of 15 patients with biopsy-confirmed CA, and no uptake in patients without CA [17]. Another study reported significantly greater myocardial [^11^C]PiB uptake in patients with AL-CA (*n* = 41) compared to 14 healthy subjects [17]. Further research involving 36 patients with AL-CA, 21 with ATTR-CA, and 15 with non-amyloid left ventricular (LV) hypertrophy or healthy volunteers revealed the diagnostic accuracy of [^11^C]PiB uptake visual inspection to be 100% in differentiating CA patients with increased wall thickness from controls. This study also suggested that [^11^C]PiB could detect early-stage CA, as some patients with amyloidosis but no known cardiac involvement, defined as LV wall thickness > 12 mm in the absence of other cause of hypertrophy, exhibited [^11^C]PiB [18]. Additionally, [^11^C]PiB uptake was significantly higher in AL-CA than in ATTR-CA: at 10 to 20 min after [^11^C]PiB injection, AL patients had the highest standardized uptake value (SUV) (2.61) and retention index (RI) (0.086 min^−1^), followed by ATTR patients (SUV 1.64, RI 0.045 min^−1^) and controls [18].

In another study with 47 patients (17 AL, 22 ATTRv, 8 ATTRwt), a combined approach of [^11^C]PiB PET and [^99m^Tc]PYP scintigraphy allowed accurate differentiation between AL- and ATTR-CA. All patients with AL-CA exhibited positive [^11^C]PiB and negative [^99m^Tc]Tc-PYP uptake (“PiB pattern”), and all patients with ATTRwt-CA showed the opposite pattern (“PYP pattern”) [19, 20]. Myocardial [^11^C]PiB uptake also correlated with the amount of amyloid deposits on histology, evaluated as the percentage of amyloid-positive area in amyloid P immunohistochemistry slides [17].

Furthermore, native T1 mapping values positively correlated with target-to-background ratio (TBR) max values (considering a cut-off of 1.09; sensitivity 92.3% and specificity 100%) in CA and non-CA patients who underwent cardiac [^11^C]PiB PET/MRI [21].

#### Risk prediction

Myocardial [^11^C]PiB uptake independently predicted clinical outcomes in AL-CA, with patients having the highest PiB uptake experiencing the shortest event-free survival [17]. In a prospective study on 58 patients with AL-CA, myocardial [^11^C]PiB uptake emerged as a strong independent predictor of 1-year overall mortality and refined risk prediction beyond the traditional AL-CA staging system, based on commonly used serum biomarkers, such as troponin I, N-terminal pro-B-type natriuretic peptide, and the difference between free light chains [22]. Although some other reports have suggested a potential role for [^11^C]PiB PET in risk stratification [23], larger-scale studies are necessary to confirm these findings.

#### Monitoring

In a case report by Fujioka et al., [^11^C]PiB PET/CT was employed to monitor a patient on tafamidis and demonstrated a reduction in amyloid deposition after almost 1 year of treatment [24]. Although the tracer uptake correlates with the degree of amyloid deposition, there are no studies on AL-CA exploring the potential application of [^11^C]PiB PET scans in determining whether amyloid clearance is correlated with clinical response [25].

### ^18^F-flutemetamol

#### Chemical structure, half-life, and FDA approval

[^18^F]Flutemetamol is structurally similar to [^11^C]PiB but features a 3’ [^18^F] fluorine substitution [26]. [^18^F]Flutemetamol has a half-life of 110 min, allowing its distribution to PET centers without on-site cyclotrons [27]. It is the second ^18^F-labeled PET radiopharmaceutical approved by the Food and Drug Administration (FDA) for in vivo detection of amyloid deposits in 2013 [28]. The first report of myocardial retention of [^18^F]Flutemetamol in CA (specifically in AL-CA) was published in 2014 [29].

#### Diagnosis

**Fig. 2:**
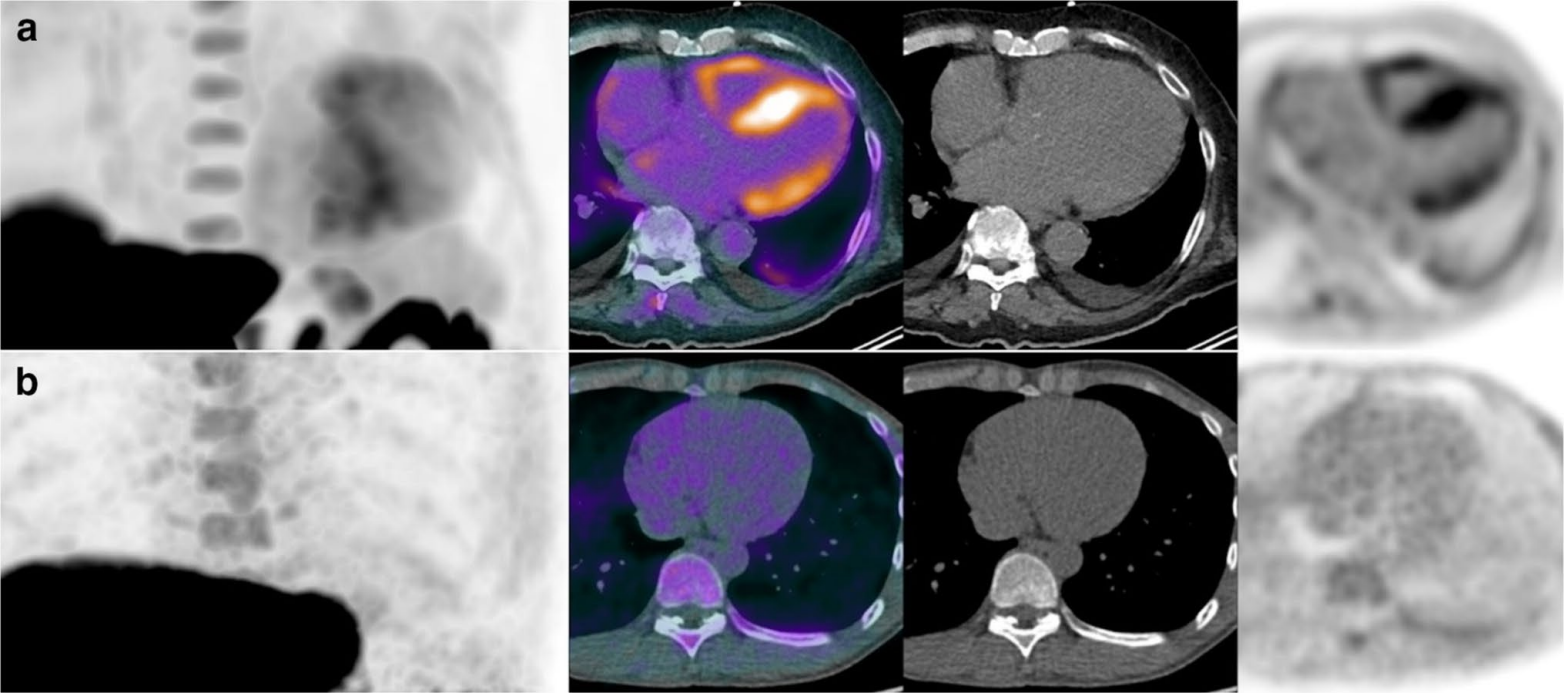
[^18^F]Flutemetamol imaging in subject with cardiac amyloidosis. **a** Showing high radiotracer uptake in the left and in the right myocardium, versus a representative control. **b** with no significant uptake in the myocardium, including in columns from the left to the right: maximum intensity projections (MIP), fused positron emission tomography (PET)/computed tomography (CT), CT, and PET. Reprinted with permission from [30]

A 2019 pilot study assessing the diagnostic properties of [^18^F]Flutemetamol PET in CA included 9 CA patients (8 with ATTR and 1 with AL) and 3 controls without CA. The study observed [^18^F]Flutemetamol uptake in 8 of the 9 CA patients and none of the controls during a 30-min list-mode acquisition, a data collection technique in which detector counts are stored sequentially as scan time progresses. The median TBR was significantly higher in CA patients than controls, with the single AL-CA patient showing a higher TBR than those with ATTR-CA (1.46, IQR 1.32–2.06 versus 1.06, IQR 0.72–1.1, *p* = 0.033) [30] (Fig. 2). In 2020, a study involving 21 patients with V30M ATTRv-CA and 6 controls (5 AD patients and one healthy individual) evaluated ^18^F-flutemetamol uptake over a 60-min dynamic PET acquisition. This study found 88% sensitivity and 100% specificity in identifying ATTRv-CA patients at 30- or 60-min static image acquisitions. The authors proposed a SUV cut-off of 1.46 for the intraventricular septum at 30 min as a diagnostic cut-off [31]. Another retrospective study investigated [^18^F]Flutemetamol uptake in 12 CA patients (7 ATTRwt, 3 ATTRv, 2 AL) and 5 with non-amyloid heart failure, with acquisition times set at 60 to 90 min post-injection. The study found no significant difference in quantitative tracer uptake between the two groups, and increased uptake was observed in only 2 of the 12 amyloidosis patients, suggesting that late acquisition is not sensitive for detecting cardiac amyloid deposits [32].

#### Risk prediction and monitoring

Further research is necessary to establish the specific acquisition protocol and the diagnostic accuracy of [^18^F]Flutemetamol in CA, as well as to explore the potential of its use in prognostic stratification and the assessment of therapeutic response, as in an ongoing trial (NCT05374564) conducted on 12 patients comparing the disease burden at 0 and 6 months of treatment with tafamidis.

Cyano-flutemetamol is an [^18^F]Flutemetamol analog with the addition of a cyano group, allowing to assess tracer binding to amyloid on histology. It binds amyloid deposits with a good agreement with Congo red and [^11^C]PiB, and a greater affinity for ATTR than AL amyloid. In a post-mortem histofluorescence analysis, the density of cyano-flutemetamol displayed a robust correlation with LV septal and posterior wall thickness, and LV mass [25].

### [^18^F]Florbetapir

#### Chemical structure, half-life, and FDA approval

Similar to the other [^18^F] radiolabeled tracers, the longer half-life (110 min) of this aromatic ether represents a huge advantage respect to [^11^C]PiB [33]. In 2012, the FDA approved the first β-amyloid imaging PET probe for the in vivo detection of amyloid deposits in AD, as it has a high affinity for β-amyloid [34, 35].

#### Diagnosis

The possibility to discriminate CA from healthy controls emerged from an autoradiography study performed on autopsy samples from AL, ATTR-CA, and controls [36] and in a pilot study conducted on 9 CA patients (5 AL, 4 ATTR) and 5 controls (3 healthy subjects and 2 with non-ischemic heart failure) [37]. Using a 60-min scan duration, the LV RI, TBR, myocardial SUV, and myocardial-to-liver SUV were all higher in CA patients compared to controls. A trend towards a higher RI was observed in AL-CA patients compared to those with ATTR-CA [37]. Osborne et al. found that the uptake time before the acquisition, the length of the acquisition, and the window/level setting all significantly impacted the capability to detect and identify the differences between controls and disease populations; so, they developed a 20-min list-mode protocol with acquisition windows at 0–5, 10–15, or 15–20 min that can be easily reproduced by any trained imaging specialist at any PET center [38].

#### Risk prediction

LV amyloid burden measured by [^18^F]Florbetapir PET/CT is a powerful predictor of cardiovascular events in AL amyloidosis, according to the 2012 Mayo stage, as the link between amyloid burden and cardiovascular events was mainly mediated by NT-proBNP, a component of Mayo stage [39]. Interestingly, [^18^F]Florbetapir PET/CT could detect early right ventricular (RV) amyloid in systemic AL amyloidosis before the occurrence of changes in RV structure and function, and predicts cardiovascular events [40].

#### Monitoring

A small study of 15 patients assessed the possible use of [^18^F]Florbetapir PET to monitor AL-CA progression before and after chemotherapy. The study observed greater cardiac uptake in chemotherapy-naïve patients compared to those already undergoing chemotherapy (RI 0.21 vs. 0.14 min^−1^), and in patients without at least a partial hematological response (RI index 0.2 vs. 0.14 min^−1^). To date, no correlations between changes in cardiac uptake, cardiac biomarkers, or serum free light-chains were noted, but larger studies are needed to explore the potential role of this tracer [41].

### [^18^F]Florbetaben

#### Chemical structure, half-life, and FDA approval

[^18^F]Florbetaben, an ^18^F-labeled stilbene derivative with a high affinity for brain β-amyloid, shares structural features with [^11^C]PiB, and has a half-life of 110 min. It was approved by FDA in 2014 [42].

#### Diagnosis

In a pilot study, 10 CA patients (5 ATTR and 5 AL) and 4 hypertensive heart disease controls were examined. All patients with AL- or ATTRwt-CA had histological disease confirmation. LV SUV was measured on static images taken between 5 and 10 min after injection. The study reported increased myocardial uptake in all CA patients, with significantly higher TBR and SUV values, in particular for AL-CA patients. This study also suggested a correlation between tracer retention and left ventricular ejection fraction on echocardiography [43]. The diagnostic value of [^18^F]Florbetaben PET was confirmed in a study assessing 9 patients with known or suspected CA (7 with biopsy-proven systemic AL amyloidosis, 5 with known cardiac involvement). The authors reported an intense myocardial uptake in patients with biopsy-confirmed CA, whereas those without CA showed low myocardial uptake [44]. A 2019 study by Kircher et al. with 22 patients (12 AL, 5 ATTR, 2 AA) suggested a role for [^18^F]Florbetaben PET in distinguishing between AL- and ATTR-CA [45]. PET images were dynamically acquired for 30 min post-injection, with static reconstructions between 10 and 30 min for visual evaluation. This study introduced the idea of discriminating between AL- and ATTR-CA through semi-quantitative evaluation of radiotracer uptake, evaluated at a later time interval than the previous pilot study [43]. Indeed, in 30 min of total imaging duration, myocardial tracer retention (MTR) values were significantly higher in AL-CA patients (median MTR of 66 with a range of 38–111) than those with ATTR-CA (median MTR of 42 with a range of 38–45, *p* < 0.01) and controls (median MTR of 27, under the detected cut-off of 36 to distinguish CA from non-CA), and correlated well with echocardiographic parameters, LV wall thickness (*r* = 0.46, *p* < 0.05) and apical sparing of the longitudinal strain pattern (*r* = 0.52, *p* < 0.02) [45].

**Fig. 3:**
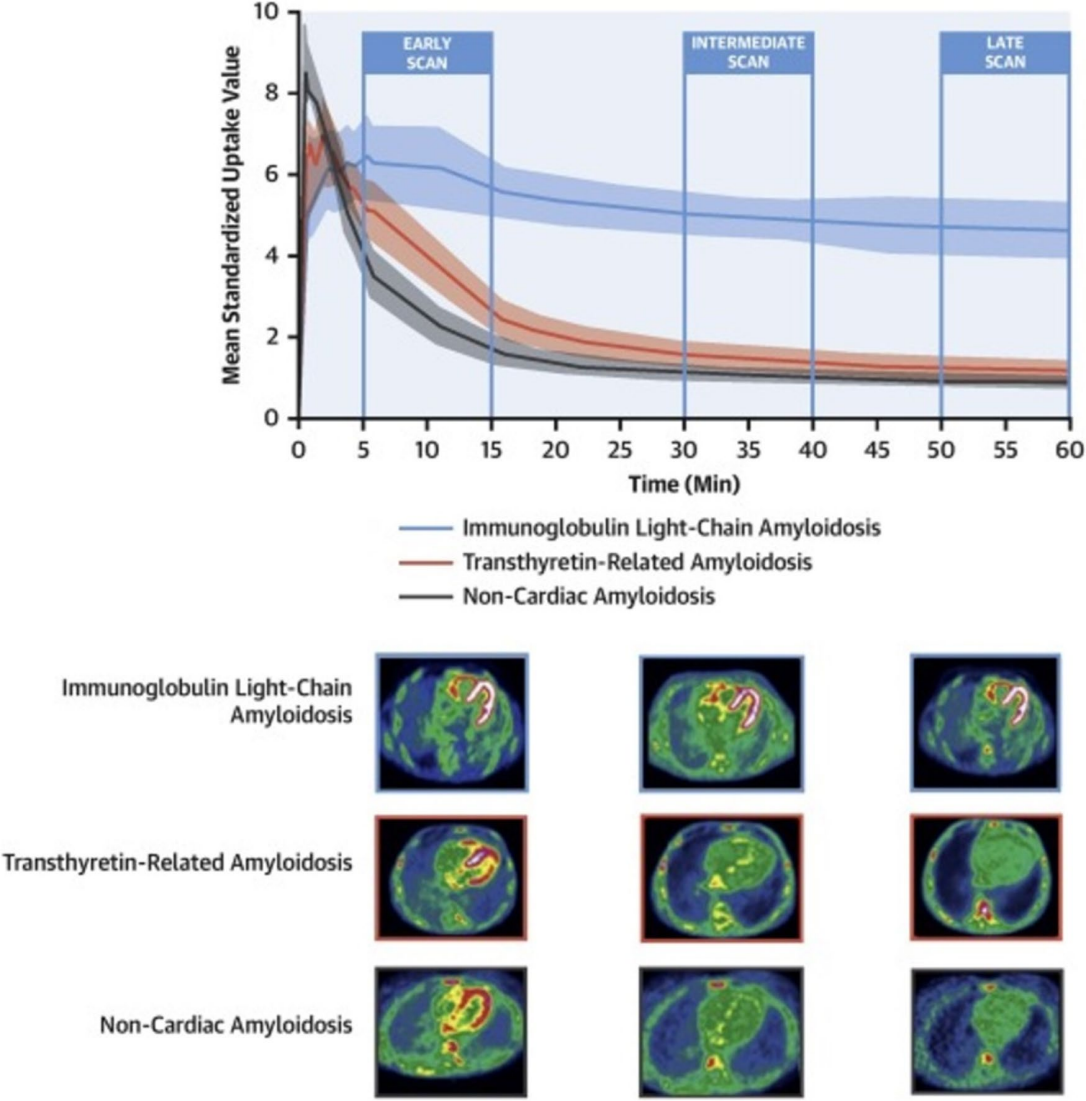
[^18^F]Florbetaben positron emission tomography for the differential diagnosis between cardiac amyloid subtypes and mimicking conditions. (Upper panel) Time–activity curves for myocardial uptake in cardiac positron emission tomography using [18F]Florbetaben are shown for patients with immunoglobulin light-chain amyloidosis (AL) (blue), transthyretin amyloidosis (ATTR) (red), and non-cardiac amyloidosis (non-CA) (gray). Shaded areas indicate the 95% confidence interval for each curve. (Lower panel) Early-phase (5–15 min), intermediate-phase (30–40 min), and late-phase (50–60 min) [18F]-florbetaben PET scans are displayed for patients with AL and ATTR cardiac amyloidosis (CA) and non-CA. SUVmean represents the mean standardized uptake value. Reprinted with permission from [46]

In 2020, Genovesi et al. conducted a study on 40 CA patients (20 AL and 20 ATTR) and 20 with LV hypertrophy. They performed dynamic PET scans from tracer injection for 60 min and delayed static scans at approximately 110 min. This delayed acquisition allowed to reliably distinguish AL-CA (early SUV 5.55; delayed SUV 3.50) from ATTR-CA (early SUV 2.55; delayed SUV 1.25) or LV hypertrophy (early SUV 3.50; delayed SUV 1.40) (Fig. 3) [46].

Then, Santarelli et al. evaluated 21 CA patients (11 AL and 10 ATTR) and 15 controls with non-amyloidotic cardiomyopathy. This study confirmed that [^18^F]Florbetaben affinity for amyloid substance is greater in AL-CA [47]. The same authors then investigated deep-learning tools to characterize CA deposits by evaluating static images acquired early (15 min) after radiopharmaceutical injection. The study included 47 subjects (13 ATTR-CA, 15 AL-CA, 19 non-amyloidotic cardiomyopathy), finding high accuracy, sensitivity, and specificity in distinguishing CA from non-CA and AL- from ATTR-CA [48]. Further studies on larger populations are needed to confirm the minimum time required from tracer injection to acquisition and to validate the utility of deep-learning techniques in assisting human interpretation.

#### Risk prediction and monitoring

The prognostic significance of the tracer remains to be explored. Vergaro et al. recently explored the prognostic significance of [^18^F]Florbetaben uptake in 40 patients with AL-CA: a late LV total amyloid burden ≥ 273 cm^3^ and late right ventricle total amyloid burden ≥ 135 cm^3^ predicted 18 and 24-month all-cause mortality independently from Mayo stage [49]. The ongoing phase 3 trials PETAL (NCT06048601) and CARdiag (NCT05184088) are investigating the diagnostic performance for visualizing and quantifying amyloid in patients with suspected AL-CA.

### ^18^F-sodium fluoride

#### Chemical structure, half-life, and FDA approval

Because of the better properties of ^99m^Tc-labeled molecules such as half-life major than the one of [^18^F]-radiolabeled tracers, they replaced one of the earliest radiopharmaceuticals, [^18^F]-NaF, for imaging applications in the 1970s. Despite approval in 1972 for use in bone scans, the ionic compound comprised of a single sodium atom bound to a positron-emitting isotope of fluorine [^18^F]-NaF was withdrawn in 1975. In the 1990s, advancements in PET scanners enabled better imaging, leading to its return in 1993 and FDA approval in 2000 [50].

#### Diagnosis

The diagnostic value of [^18^F]-NaF PET has been explored based on the notion that different amyloid proteins impact calcium homeostasis. In 2016, Van Der Gucht for the first time demonstrated in two patients that [^18^F]-NaF PET/CT could differentiate ATTR-CA from AL-CA with a faster kinetics and then imaging time than ^99m^Tc-labeled diphosphonates: [^18^F]-NaF images showed a diffuse myocardial uptake in the ATTR-CA patient in the early phase, more than in the delayed phase, reflecting a faster wash-out; the AL-CA patient had no myocardial uptake at both acquisition times, indicating a different affinity for the tracer [51]. In recent studies, including two series each comprising 7 CA patients (one with 5 ATTR-CA + 2 AL-CA, the other 4 ATTR-CA + 3 AL-CA), the authors confirmed higher myocardial [^18^F]-NaF uptake in ATTR-CA compared to AL-CA [52, 53]. The first series utilized PET/CT 1 h post-injection, revealing a 58% higher mean SUV over the entire myocardium in ATTR-CA than in AL-CA patients, with variations in uptake intensity across myocardial segments [52]. The second series employed PET/MRI with a dynamic acquisition over 90 min starting 5 min post-injection, finding myocardial TBR values 48% higher in ATTR CA than in AL CA patients, exhibiting a patchy pattern of uptake [53]. In a larger cohort with suspected CA, [^18^F]-NaF PET/MRI showed its capability to distinguish ATTR from AL amyloidosis and non-amyloid patients, particularly when myocardium to blood pool ratio semi-quantification was used, as visual interpretation displayed lower contrast [54].

**Fig. 4:**
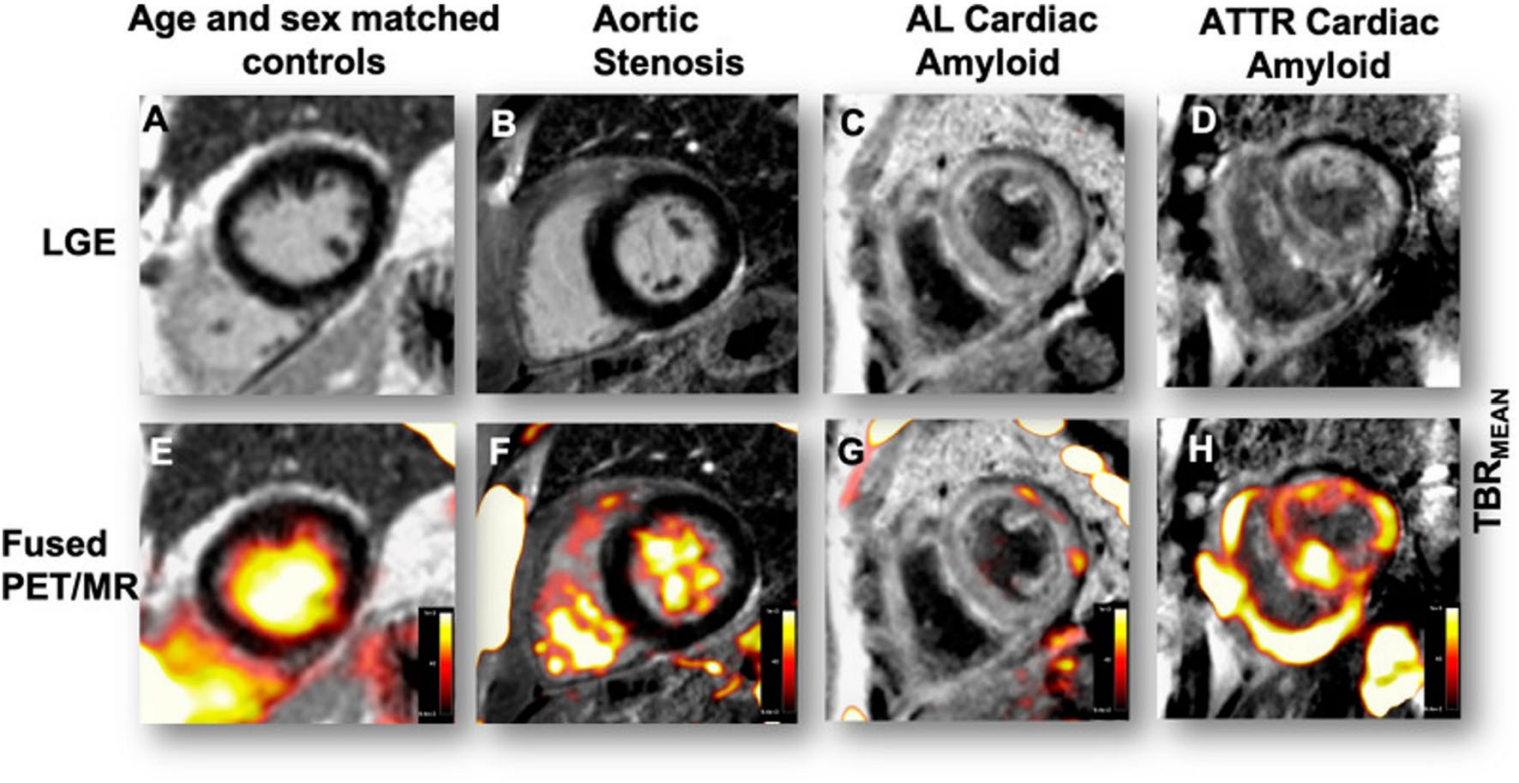
Patterns of [^18^F]-fluoride uptake between cohorts. Columns represent each cohort and rows imaging modality in the short axis view. Panel **A** shows a delayed enhanced image of a control subject with normal myocardial mass and no late godolidium enhancement (LGE). The corresponding fused positron emission tomography/magnetic resonance (PET/MR) image (**E**) shows uptake only in the blood pool. Panel **B** is a patient with aortic stenosis and elevated left ventricular mass. Note the absence of myocardial.^18^F-fluoride uptake on panel **F** and similar to the healthy control, uptake is greater in the blood pool than myocardium. Panel **C** shows a patient with light-chain (AL) amyloid displaying the characteristic myocardial nulling difficulties with LGE found in cardiac amyloidosis. Panel **G** shows patchy lateral wall uptake greater than the blood pool. Panel **D** shows similar LGE findings, but this time in transthyretin (ATTR) amyloid. Note the striking and extensive biventricular uptake in panel **H**, much greater than the blood pool and what was seen in AL. Target-to-background ratio (TBR). Reprinted with permission from [56]

A study found a significantly higher TBR in ATTR-CA (*n* = 7) (0.98 ± 0.09) as compared to AL-CA (0.85 ± 0.08; *p* = 0.026) and controls (0.82 ± 0.07; *p* = 0.020), suggesting that a TBR cut-off of 0.89 could discriminate the 2 types of CA [55]. In a study of quantitative ^18^F-fluoride PET/magnetic resonance imaging (MRI), TBR was significantly higher in patients with ATTR-CA (*n* = 10) (1.13 ± 0.16) than healthy controls (0.84 ± 0.11, *p* = 0.0006) and other similar phenotypes, such as patients with aortic stenosis (0.73 ± 0.12; *p* < 0.0001) or AL-CA (0.95 ± 0.08; *p* = 0.01). Moreover, linking TBR PET-findings with late gadolinium enhancement on cardiac MRI could get higher specificity and sensitivity to distinguish ATTR-CA and AL-CA [56] (Fig. 4). Despite these promising findings, [^18^F]-NaF PET/CT remains inferior in terms of sensitivity with a value of 0.25 (95% CI 0.089 to 0.53) compared to [^99m^Tc]Tc-PYP single-photon emission computerized tomography (SPECT) (100%, *p* = 0.016) for the diagnosis of ATTR-CA [57].

#### Risk prediction and monitoring

To date, no data is available on the use of tracers for prognosis definition and therapy response monitoring.

### [^124^I]Evuzamitide

#### Chemical structure, half-life, and FDA approval

Evuzamitide, also known as p5 + 14, is a pan-amyloid-reactive peptide, binding all forms of amyloid, included AL kappa, AL lambda, ATTRv, leukocyte chemotactic factor-2, apolipoprotein-A2c, serum amyloid A, and islet amyloid polypeptide, derived from various anatomic sites, overcoming the problem of the etiological heterogeneity of this systemic disease [58]. In August 2024, the FDA granted its breakthrough therapy designation to the PET [^124^I]Evuzamitide for imaging in patients with suspected or known CA. The completion of the phase 3 REVEAL study (NCT06788535) evaluating the efficacy and safety of [I-124]Evuzamitide is estimated for the end of 2025. This peptide labeled with iodine-124 is a cyclotron-produced radionuclide with a 4.2-day half-life.

#### Diagnosis

**Fig. 5:**
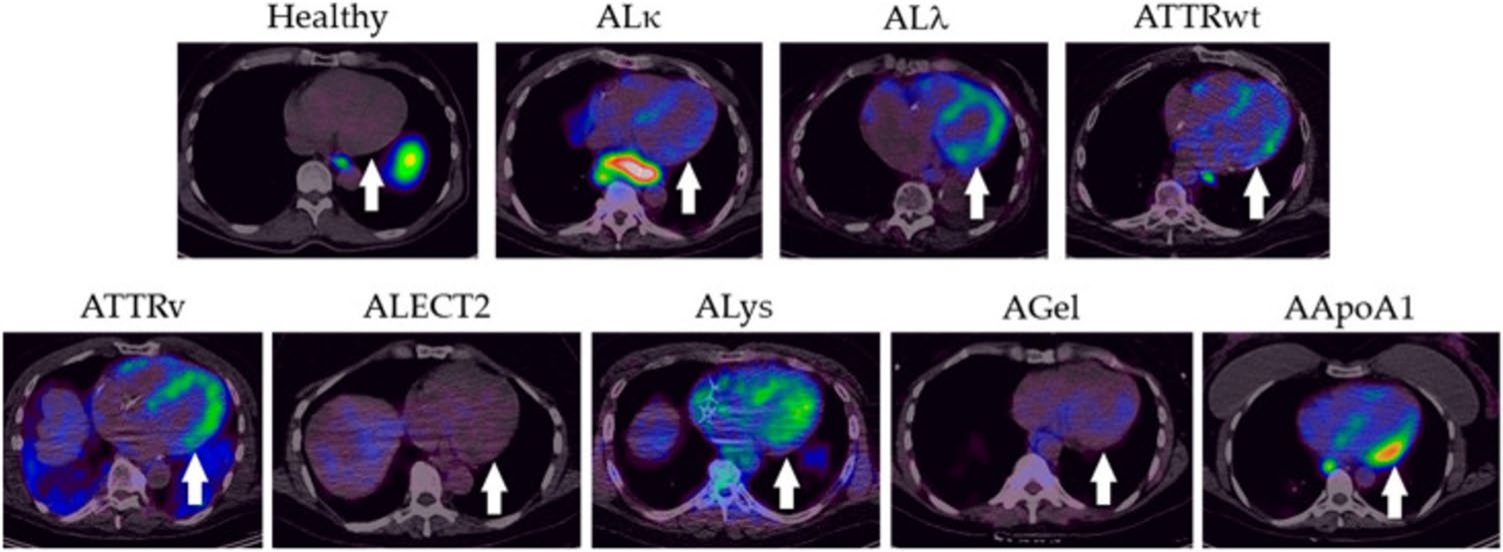
Transaxial images of the heart from a representative healthy subject and patients with diverse forms of systemic amyloidosis following injection of [.^124^I]Evuzamitide. Retention of radiotracer in the left ventricular wall (arrow) was seen in all subjects except the healthy individual and patients with leucocyte chemotactic factor 2 amyloidosis (ALECT2) and gelsolin amyloidodosis (AGel). ALκ, kappa light chain amyloidosis; ALλ, lambda light chain amyloidosis; Alys, lysozyme amyloidosis; AApoA1, apolipoprotein-A1 amyloidosis; ATTRv, mutated/variant transthyretin amyloidosis; ATTRwt, wild-type transthyretin amyloidosis. Reprinted with permission from [58]

A single-site, open-label first-in-human phase 1/2 study (NCT03678259) substantiates the general safety and effectiveness of [^124^I]Evuzamitide—in identifying cardiac and systemic amyloid deposits with high sensitivity (93.6%) in 50 patients with systemic amyloidosis, 2 asymptomatic ATTRv carriers, and 5 healthy volunteers [59]. As patients involved in this study presented different types of systemic amyloidosis, [^124^I]Evuzamitide PET showed different patterns of cardiac uptake: although right ventricular and atrial walls were also observed in certain patients with AL, ATTR, lysozyme, and apolipoprotein-A1 amyloidosis, the LV, particularly the interventricular septum and posterior wall, was the primary site of myocardial uptake of [^124^I]Evuzamitide; then, only trace blood pool activity was observed in the ventricular lumen in patients with leukocyte chemotactic factor-2, gelsolin, and healthy people, suggesting no amyloid deposits in the myocardium [58] (Fig. 5). A pilot study including 46 participants (12 AL-CA, 12-ATTRwt, 2 ATTRv, and 20 controls without CA) compared [^18^F]Florbetapir and [^124^I]Evuzamitide, finding a comparable discrimination performance for the two tracers in AL-CA, and a preferable application of [^124^I]Evuzamitide in ATTR-CA. Moreover, this study revealed the correlation between the radiotracer uptake and morphologic and functional cardiac parameters measured with echocardiogram and MRI, such as interventricular septum thickness (Spearman’s *ρ* = 0.78), LV global longitudinal strain (*ρ* = 0.54), LV mass index (*ρ* = 0.82), and extracellular volume (*ρ* = 0.51) [60].

#### Risk prediction

This moderate-to-strong correlation suggested the potential role in the quantification of amyloid burden [60], but further research is needed to define the prognostic value of PET with this tracer.

#### Monitoring

An AL amyloidosis patient underwent a [^124^I]Evuzamitide PET/CT monitoring during treatment with daratumumab and this radiotracer allows to follow the organ response to plasma cell immunotherapy [61].

### A possible diagnostic algorithm including PET imaging

The diagnosis of CA is often delayed due to its variable clinical presentation and limited awareness. Recent advances in multimodality cardiovascular imaging, such as transthoracic echocardiography-derived speckle tracking imaging, MRI, and radionuclide imaging techniques, have revolutionized the non-invasive diagnosis of CA [62, 63]. While echocardiography and MRI predominantly provide structural and functional data, they cannot accurately discriminate between ATTR- and AL-CA. Achieving a timely definite diagnosis is essential to guide appropriate treatment, whether targeted therapies for ATTR-CA or chemotherapy and immunotherapy regimens for AL-CA.

**Fig. 6:**
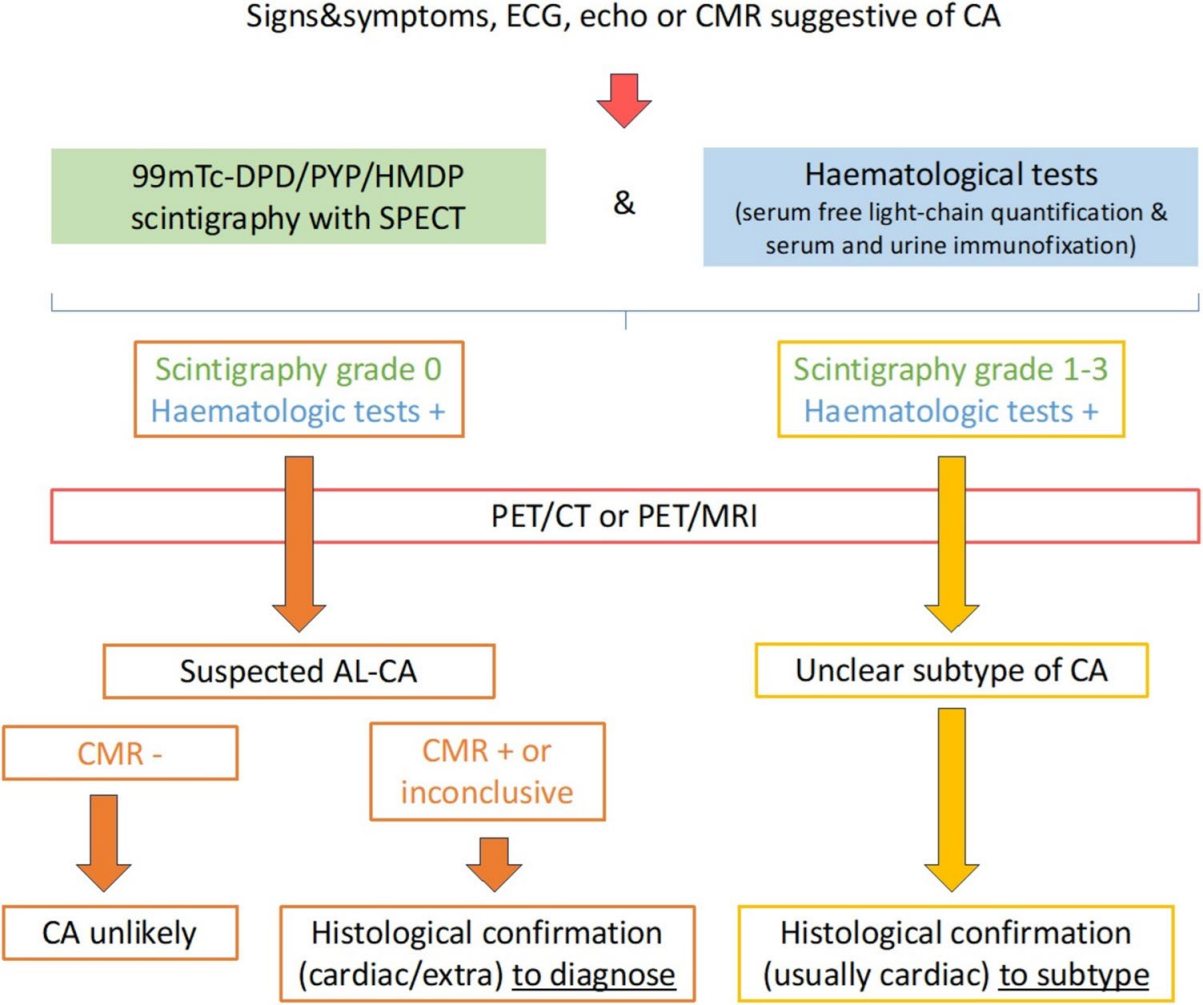
Possible integration of positron emission tomography (PET) in the diagnostic workup of cardiac amyloidosis. AL, light chain amyloidosis; ATTR, transthyretin amyloidosis; CA, cardiac amyloidosis; CT, computed tomography; CMR, cardiovascular magnetic resonance; ECG, electrocardiogram; MRI, magnetic resonance imaging

PET imaging could be extremely valuable for an early, non-invasive diagnosis of CA [62, 64]. Planar scintigraphy, using bone tracers such as [^99m^Tc]-labeled diphosphonates, plays a pivotal role in diagnosing ATTR-CA, and is included in the diagnostic algorithm for the diagnosis of amyloidosis, which is provided in the position statement of the European Society of Cardiology (ESC) Working Group [9]. However, the technique has some critical issues, such as false negatives that occur in the case of some variants of ATTR-CA or in the case of AL-CA. Nonetheless, scintigraphy lacks specificity for ATTR-CA, as it can also yield positive results in cases of AL-CA. This underscores the necessity of a tissue biopsy in the presence of a Perugini score of 1 and/or a monoclonal component to achieve diagnostic certainty. To solve these problems, the use of pan-amyloid tracers, such as evuzamitide, offers the potential to localize amyloid deposits throughout the body and monitor treatment response through repeated scans. Moreover, emerging data indicate that [^18^F]Florbetapir [65] and [^11^C]PiB PET [18] can diagnose early AL-CA, even before significant LV wall thickening or cardiac biomarker release. The preliminary evidence described above [46] lays the foundation for ongoing studies regarding the potential diagnostic value of [^18^F]Florbetaben (NCT06048601 and NCT05184088). If the good diagnostic performance of [^18^F]Florbetaben PET in patients with suspected CA and positive serum/urine immunofixation, PET may bypass the need for tissue biopsy in the presence of a monoclonal component (Fig. 6).

## Conclusions

**Fig. 7:**
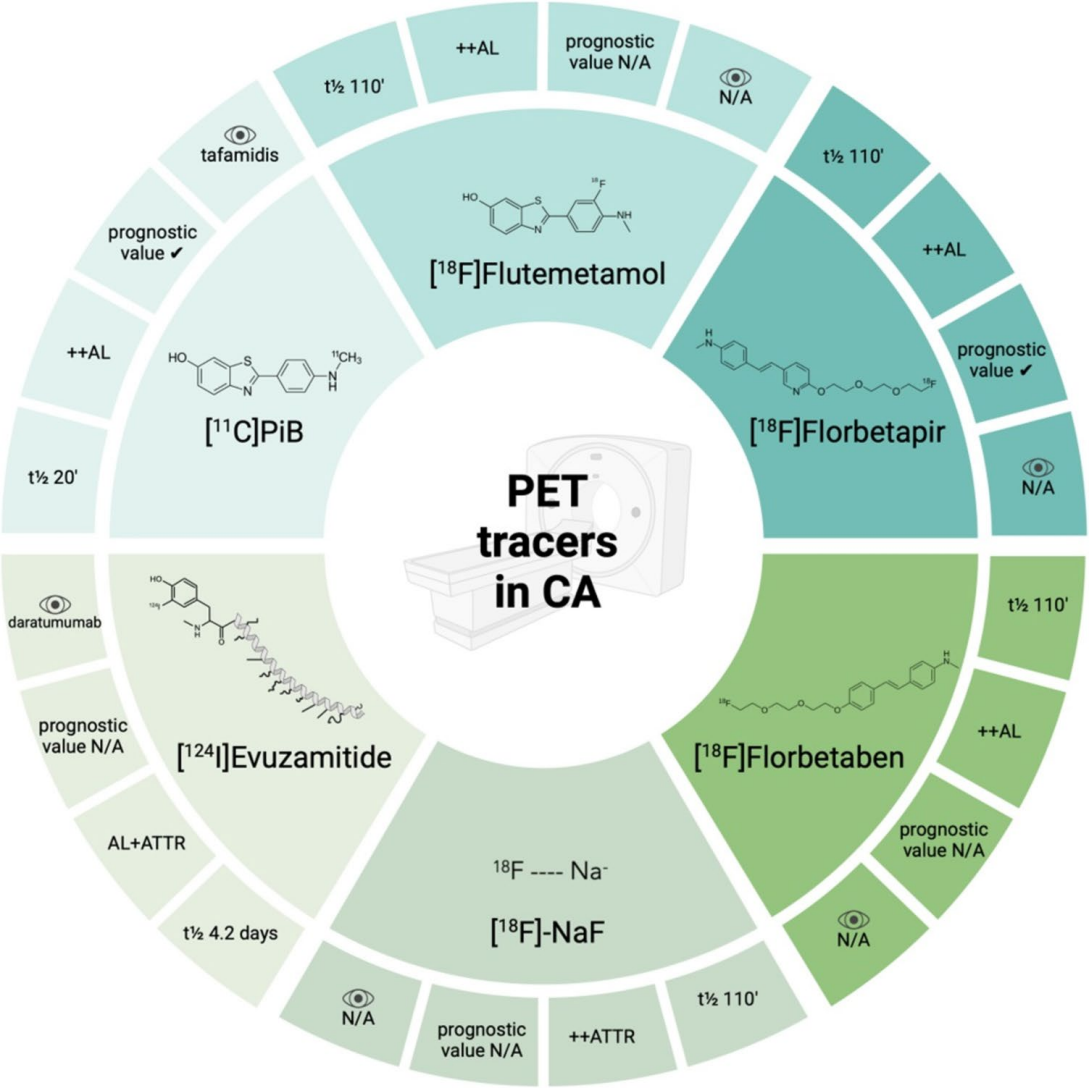
Central illustration. Positron emission tomography tracers in cardiac amyloidosis. For each radiotracer, there is the definition of four characteristics: half-life, preference in affinity for light-chain o transthyretin amyloid, prognostic value, and utility in monitoring therapy response (eye symbol). AL, light chain amyloidosis; ATTR, transthyretin amyloidosis; CA, cardiac amyloidosis; [^11^C]PiB, [^11^C]Pittsburgh compound B; [^18^F]-NaF, ^18^F-sodium fluoride; N/A, no available data; PET, positron emission tomography; t½, half-life

The properties and proposed applications of amyloid-binding radiotracers are summarized in Table 1 (Fig. 7). Unlike bone tracers, these radiotracers, except for [^18^F]-NaF and [^124^I]Evuzamitide, demonstrate a higher affinity for AL amyloid and present a novel approach to differentiating amyloidosis subtypes, potentially eliminating the need for tissue biopsy. To date, no PET studies have investigated rare types of CA, such AA or ALECT2 amyloidosis, predominantly involving extra-cardiac organs. PET imaging also enables the quantification of disease burden, offering more than just a semi-quantitative evaluation of cardiac involvement, as with scintigraphy. Scientific societies have proposed various measures achievable with planar scintigraphy to integrate the standard criterion (Perugini 2–3) for non-invasive diagnosis.

**Table 1:** Characteristics of the proposed tracers for positron emission tomography

	[^11^C]PiB	[^18^F]Flutemetamol	[^18^F]Florbetapir	[^18^F]Florbetaben	[^18^F]-NaF	[^124^I]Evuzamitide
Development	Initially used to quantify β-amyloid in AD (2002) [15]	FDA approval in 2013 [28]; the first report of use in AL-CA in 2014 [29]	The first PET tracer approved by FDA (2012) for AD [34]	Approved by FDA in 2014 [42]	First FDA approval in 1972 for bone scans; withdrawal in 1975; approved in 2000 [50]	FDA’s breakthrough designation for CA imaging in 2024
Tracer properties	• Short t½ (20.4’) [14] • Need for on-site cyclotron	• Long t½ ([27] 110’) • Late acquisition is not sensitive [32] • Correlation with Congo red/[^11^C]PiB uptake [25]	• Long t½ (110’) [33] • Availability for brain imaging [14]	Long t½ (110’) [14]	• Long t½ (110’) [14] • Faster kinetics and imaging time [51, 52]	• t½ 4.2 days [58]
Diagnostic applications	• Detecting either AL- or ATTR-CA [16] • Detecting early-stage CA [18]	• High sensitivity (88%) and specificity (100%) for ATTRv-CA [31] • Correlation with LV wall thickness, and heart mass [25]	• High LV RI, TBR, SUV in AL-/ATTR-CA [37, 40] • Early identification of RV amyloid in AL-CA [36] • Promising role in the diagnosis, prognostication, and monitoring therapy response in ATTR-CA [41]	• High TBR, SUV in AL-/ATTR-CA [43] • Intense myocardial uptake in CA [44] • Correlation with echo and MRI parameters [43, 45]	• Inferior sensitivity than gold standard [^99m^Tc]Tc-PYP SPECT [57]	• Pan-amyloid tracer [58] • Accurate discrimination of AL-/ATTRwt-CA from controls [60] • Correlation with echo and MRI parameters [60]
Use for differential diagnosis (AL- vs. ATTR-CA)	• AL-CA > ATTR-CA [18] • AL-CA: positive [^11^C]PiB and negative [^99m^Tc]Tc-PYP uptake (“PiB pattern”). ATTRwt-CA: the opposite (“PYP pattern”) [19, 20]	• AL-CA > ATTR-CA [30] • Cyano-flutemetamol ATTR-CA > AL-CA [25]	AL-CA > ATTR-CA [37]	• AL-CA > ATTR-CA [43] • AL-CA > ATTR-CA and controls in terms of MTR [45] and SUV [46]	• ATTR-CA > AL-CA • Diffuse uptake in the early > delayed phase in ATTR-CA; no uptake in AL-CA [51, 52] • TBR for ATTR-CA > AL-CA [55, 56]	• ATTR-CA > AL-CA • Discrimination performance like [^18^F]Florbetapir in AL-CA; better performance in ATTR-CA [60]
Prognostic value	• Quantification of amyloid burden [17] • Prognostic value in AL-CA [17, 22]	N/A	• Quantification of amyloid burden [67] • Prognostic value in AL-CA [39] • No correlation with biomarkers or free light chains [43]	LV amyloid burden ≥ 273 cm^3^ and late RV amyloid burden ≥ 135 cm^3^ predicted 18 and 24-month all-cause mortality independently from Mayo stage [49]	N/A	N/A
Monitoring response to therapy	Monitoring tafamidis response in ATTR-CA[24]	N/A	Potential role in monitoring chemotherapy response in AL-CA [41]	Potential role in monitoring therapy response in AL/ATTR-CA [43]	N/A	Monitoring daratumumab response in AL-CA [61]
Open issues	Prognostic accuracy [23]	Acquisition protocol, assessment of treatment response	Assessment of treatment response	Prognostic accuracy, assessment of treatment response	Prognostic accuracy, assessment of treatment response	Prognostic accuracy

*AD*, Alzheimer’s disease; *AL*, light-chain amyloidosis; *ATTR*, transthyretin amyloidosis; *ATTRv*, mutant/variant transthyretin amyloidosis; *ATTRwt*, wild-type transthyretin amyloidosis; *CA*, cardiac amyloidosis; *[*^*11*^*C]PiB*, [^11^C]Pittsburgh compound B; *[*^*18*^*F]-NaF*, [^18^F]-sodium fluoride; *FDA*, Food and Drug Administration; *LV*, left ventricle; *MTR*, myocardial tracer retention; *N/A*, no available data; *PYP*, pyrophosphate; *RI*, retention index; *RV*, right ventricular; *SUV*, standardized uptake value; *t½*, half-life; *TBR*, target-to-background ratio; *wt*, wild-type

The ESC and the German Cardiology Society (DKG) have proposed Perugini score ≥ 2 on a 99mTc-DPD or [^99m^Tc]Tc-PYP scan after 3 h; the American societies propose Perugini score ≥ 2 and/or a heart-contralateral ratio (H/CL) ratio ≥ 1.5 on a 99mTc-PYP scan after 1 or 3 h; finally, the Japanese society specifies that the H/CL must be > 1.5 on a 1-h scan or > 1.3 on a 3-h scan [66].

These proposals may represent an attempt to improve the diagnostic performance of planar scintigraphy without SPECT. Unlike these methods, PET allows quantifying the amyloid burden. For example, physicians can precisely assess the amyloid burden thanks to quantitative imaging made achievable by amyloid-specific PET tracers: total amyloid burden, calculated as mean standardized uptake value multiplied by molecular volume, was assessed in the left and right ventricles in early (5–15’) and late (50–60’) acquisitions [49]. This quantitative assessment contributes to the follow-up of the disease extension, directly proportionate to the tracer uptake, and the treatment effectiveness. Amyloid load measurement supports therapeutic decision-making by directing treatment plans and providing prognostic data [67].

Despite these advancements, we need further evidence to consolidate the role of PET in both the diagnostic algorithm and the management of patients with CA. The ^18^F-labeled tracers are subject to intensive investigation due to their extended half-life, which obviates the need for on-site cyclotrons. However, studies involving fluorine-labeled radiotracers have yielded mixed results, and the mechanisms underlying their differential binding to ATTR or AL amyloid fibrils, including potential tropism for specific fibril patterns and amyloid protein subtypes, are yet to be clarified.

There is a need to standardize PET imaging protocols for CA, particularly defining the timing of acquisition, selection of quantitative methods, and threshold values. Currently, there are various methods to measure PET tracer uptake, broadly categorized into dynamic methods like the myocardial RI, and static ones such as SUV and TBR. Notably, temporal changes in SUV have enabled differentiation between AL and ATTR disease in CA. Harmonizing and optimizing these quantitative methods are essential for consistent inter-study comparisons and for evaluating patient responses to amyloidosis-specific treatments.

PET imaging holds potential to enhance prognostication in CA and to monitor disease progression over time and across treatment regimens. Large, multi-center studies are necessary to evaluate the incremental prognostic value of PET alongside other imaging modalities in CA and to characterize and compare the diagnostic yields of different ^18^F-labeled tracers. More insight is expected from ongoing studies and the characterization of [^124^I]Evuzamitide as a pan-amyloid tracer. Integrated PET and MRI imaging might offer additional benefits in terms of diagnosis and disease monitoring in CA. Hybrid techniques are expected to play an increasing role in future studies, further advancing the field of CA diagnostics and management.

## Data Availability

No datasets were generated or analysed during the current study.
